# Perceptions about medical aid in dying among healthcare workers in Pakistan

**DOI:** 10.1017/S1478951525101284

**Published:** 2025-12-26

**Authors:** Habiba Zaheer, Muhammad Atif Waqar, Tushar Subash, Adil Elahi, Shiza Atif, Aisha Ambreen, Asra Taj, Ismat Jabeen

**Affiliations:** 1 Department of Oncology, Section of Palliative Medicine, The Aga Khan University Hospital, Karachi, Pakistan; 2 Psychiatry, Baptist Hospitals of Southeast Texas, Beaumont, TX, USA; 3 The Aga Khan University, Karachi, Pakistan

**Keywords:** Medical aid in dying (MAID), palliative care, end-of-life decisions, ethical attitudes, healthcare professionals

## Abstract

**Objectives:**

This study aims to examine the awareness, attitudes, and acceptability of medical aid in dying (MAiD) among healthcare professionals in Pakistan, a predominantly Muslim country where cultural and religious values heavily influence medical ethics and end-of-life decisions.

**Methods:**

A cross-sectional survey was conducted online among 70 healthcare professionals, including physicians, nurses, and allied health workers in Pakistan. Data were collected via a structured, self-administered online questionnaire assessing knowledge, attitudes, and willingness to participate in MAiD-related actions. Descriptive and correlational analyses were conducted to identify patterns and associations.

**Results:**

Participants demonstrated moderate knowledge about MAiD (M = 17.13, SD = 3.42) and moderate support for its legalization (M = 18.89, SD = 4.99). However, levels of negative attitudes (M = 32.21, SD = 6.11) and legal and ethical concerns (M = 24.73, SD = 3.66) were high. Behavioral willingness to engage in MAiD-related actions remained low (M = 2.42, SD = 3.38), with limited intent to assist (M = 0.39), refer (M = 0.64), or approve physician-assisted MAID (M = 0.81). A significant negative correlation emerged between knowledge and support for legalization (r = − .25, p = .037), while no significant associations were observed between knowledge and willingness to participate in MAiD. Gender and profession did not significantly influence attitudes or willingness.

**Significance of results:**

While Pakistani healthcare professionals display a conceptual understanding of MAiD, their readiness to participate remains low, primarily due to ethical, legal, and religious concerns. These findings highlight the need for creating awareness regarding MAiD and for providing culturally sensitive education, structured training in palliative care, and the development of clear legal frameworks to guide end-of-life decision-making in Muslim-majority contexts.

## Introduction

In the context of end-of-life care, medical aid in dying (MAiD) is a controversial issue that is debated worldwide and hinges on ethical considerations. It pertains to the voluntary termination of a patient’s life, performed at their request, mostly in cases of terminal and irreversible disease (Britannica 2021). For patients with end-stage organ failure or metastatic cancer, repeated hospitalizations and invasive procedures often lead to cumulative decline, extending survival time while eroding dignity, comfort, and autonomy (Martí-García et al. 2022). Bringing about MAiD, though, is far from easy. It often poses providers and families with difficult ethical challenges along with the complex legal and logistical complexities.

MAiD is a qualitatively distinct strategy from other interventions used at the end of life to reduce suffering. Palliative sedation, for instance, relieves suffering by decreasing consciousness without accelerating death, whereas MAID consists of the intentional, patient-initiated use of medications to directly and peacefully end life (Booker and Bruce 2020). Another strategy is euthanasia, where a physician directly gives life-ending medication upon request by the patient, unlike MAiD, which can allow for self-administration in some places (Güth et al. 2023). Based on Materstvedt’s study (2012), these differences have legal consequences. For example, Canada allows both MAiD and Palliative sedation but regulates them separately with an emphasis on intent over outcomes (Juth et al. 2013).

The ethical aspects of MAiD involve principles regarding autonomy, beneficence, professional integrity, and sociocultural values (Dugdale et al. 2019). Varkey, in his study, considers that 4 bioethical principles underpin MAiD discussions: autonomy (patient autonomy), beneficence (alleviating suffering), non-maleficence (aversion to harm), and justice (equitable access) (Varkey 2021). In places such as Canada, where MAiD access focuses on patient request, opponents caution unintended effects, from systemic risks of coercion to the weakening of suicide prevention norms. For example, non-maleficence can be transgressed when vulnerable patients are pressured into utilizing MAiD due to economic reasons (Christie and Li 2023). Likewise, in Islamic bioethics, the voluntary reduction of human lifespan by oneself or by others is a transgression of God’s will, creating an absolute moral limit that transcends modern concepts of individual choice in end-of-life care (Elmahjub 2022).

Legal frameworks around MAiD differ significantly across the globe. In Canada, the law permits broader eligibility, including patients who do not face imminent death. By contrast, Australia, particularly the state of Victoria, adopts a more cautious stance, requiring self-administration and restricting access (Chubb et al. 2025). European nations such as the Netherlands, Belgium, and Spain each bring their own ethical boundaries to the discussion, varying in patient age criteria, procedural types, and religious tensions (Verhofstadt et al. 2024).

Experiences of healthcare professionals regarding MAiD are not homogeneous. Some people are strongly for or against it, while others are somewhere in the middle, aligning their acceptance with specific policies, cases, or personal considerations (Stergiopoulos et al. 2024). These positions are not only legally contingent; they also reflect personal history, institutional culture, social frameworks, and emotional self-regulation (Wu et al. 2022).

Frontline workers often report a lack of clarity about defined boundaries for their roles when there is little or no institutional guidance (Fujioka et al. 2018). For clinicians in rural areas, social and geographical isolation may exacerbate moral discomfort (Steck et al. 2013). Solving these issues goes beyond teaching. It also requires defined boundaries, policies that foster inclusion, and frameworks for emotional support (Dyer et al. 2015).

Islamic jurisprudence generally prohibits euthanasia and physician-assisted suicide, grounded in the belief that life is a divine trust that humans must not terminate (Mustafa 2014). While suicide and assisted dying are categorically forbidden in Islamic teachings, scholars do make allowances in exceptional cases. For instance, when a patient is terminally ill and medical treatment offers no benefit, withholding or withdrawing care may be religiously permissible (Aramesh and Shadi 2007).

Pakistan has a special set of difficulties when it comes to providing quality palliative care. The infrastructure is woefully inadequate, with only 1 palliative care facility per 90 million people (Ali and Khokhar 2020). Due in part to societal unease with opioids and a lack of expertise, pain management is still neglected. Additionally, doctors frequently administer aggressive life-prolonging treatments even when they are unlikely to be helpful, particularly where there is a lack of explicit end-of-life laws (Faizi and Ali 2024).

The topic of MAiD is increasingly being discussed on the international level, but in Pakistan, it is given little importance. There is scant public debate on the subject, and healthcare professionals are ill-informed (Elahi et al. 2024). This is cause for concern because the importance of MAiD in palliative care as well as ethical discussions globally is on the rise (Dierickx and Cohen 2019). In Pakistan, religious sensitivities make the situation even more complex, it is important to understand how cultural background affects provider attitudes. This study is a step in this direction.

## Materials and methods

### Study design

A cross-sectional survey design was employed to examine healthcare providers’ attitudes, knowledge, and ethical concerns regarding MAiD practices

### Study setting and participants

Participants were recruited online and included physicians, nurses, nurse assistants, and physiotherapists engaged in direct patient care from varied tertiary care hospitals in Pakistan.

**Inclusion Criteria**:

- Physicians, registered nurses, nurse assistants, and physiotherapists actively employed in clinical settings within tertiary care hospitals.

**Exclusion Criteria**:

- Healthcare workers in nonclinical roles (e.g., radiology, laboratory, pharmacy).
- Undergraduate healthcare trainees.
- Individuals who did not consent to participate.

### Sample size

Initially, the planned sample size was set at 200 participants, informed by international literature, which typically ranged from 100 to 3000 participants in similar studies. However, due to logistical constraints, limited engagement in online surveys, and cultural sensitivities surrounding the topic, the final sample size obtained was 70 participants. Although smaller than anticipated, this number reflects practical limitations encountered during data collection in the local context, particularly given the sensitive and controversial nature of MAID in a predominantly Muslim country like Pakistan.

### Data collection procedure

Participants were recruited via convenience sampling through digital platforms. Data collection was conducted using a structured, self-administered online questionnaire distributed through Red Cap forms. Participants provided informed consent electronically before accessing the questionnaire.

### Study instruments and measures

The survey comprised demographic questions and validated items designed to assess:

1. **Perceptions about MAiD**: Participants’ general attitudes, beliefs, and opinions were assessed using a combination of closed-ended questions.
2. **Knowledge of MAiD**: Participants’ understanding of ethical, procedural, and legal aspects was evaluated through direct knowledge-based questions.
3. **Attitudes toward MAiD**: A Likert-scale measured personal emotional responses and beliefs concerning MAiD.
4. **Acceptability of MAiD**: Clinical scenarios and patient vignettes assessed the acceptability of MAiD under varying circumstances, including terminal illness, severe pain, or psychological distress.

### Minimizing bias

Selection bias was addressed by recruiting diverse professional roles across multiple tertiary care hospitals. Information bias was mitigated using validated survey tools with clear, straightforward language. Response bias was reduced by ensuring confidentiality and anonymous participation.

### Ethical considerations

Ethical approval was obtained from Aga Khan University’s Ethical Review Committee (AKU-ERC). Participants’ confidentiality and anonymity were maintained throughout data collection, and their participation was entirely voluntary with informed consent obtained prior to participation. This study was conducted in accordance with the ethical principles outlined in the Declaration of Helsinki.

## Results

A total of 70 healthcare professionals participated in the study, with the majority being female (64.3%) and physicians (81.4%). Participants represented a range of specialties including oncology, internal medicine, palliative care, surgery, and pediatrics. While most had over 6 years of general healthcare experience, their direct exposure to terminally ill patients was more limited, with over half reporting 10 years or less in end-of-life care.

### Perceptions toward medical aid in dying (MAID)

Participants demonstrated moderate knowledge and attitudes about MAiD (M = 17.13, SD = 3.42) and moderate support for its legalization (M = 18.89, SD = 4.99). However, negative attitudes (M = 32.21, SD = 6.11) and legal and ethical concerns (M = 24.73, SD = 3.66) were relatively high, indicating prevalent cultural, religious, and moral apprehensions.

Behavioral willingness to engage in MAiD-related actions was notably low (M = 2.42, SD = 3.38), with participants showing minimal inclination to directly assist (M = 0.39), refer (M = 0.64), or approve physician-assisted MAiD (M = 0.81). Acceptability decreased further when considering non-physician-assisted MAiD (M = 0.33), reflecting concerns regarding professional legitimacy and accountability.

A significant negative correlation was found between knowledge and support for legalization of MAID (r = − .25, p = .037), suggesting that increased familiarity with the topic may lead to more cautious or critical views. No significant relationship was observed between knowledge and willingness to engage in MAiD-related actions (r = − .17, p = .193). Furthermore, no statistically significant differences in attitudes or willingness were found based on profession or gender (p > 0.05 for all comparisons).

**Table 1.** Descriptive statistics for MAiD-related knowledge, attitudes, and behavioral intentions among participants are presented in Table 1

Variable	Mean	SD	Min	Max
Knowledge and attitude about MAiD	17.13	3.42	12	26
Support for legalization of MAiD	18.89	4.99	6	27
Negative attitude toward MAiD	32.21	6.11	22	45
Legal and ethical concerns	24.73	3.66	17	35
Willingness to engage in MAiD actions	2.42	3.38	0	12
Willingness to directly assist in MAiD	0.39	0.91	0	5
Willingness to refer for MAiD	0.64	1.16	0	4
Acceptability of physician-assisted MAiD	0.81	1.22	0	5
Acceptability of non-physician MAiD	0.33	0.66	0	3

**Table 2.** The demographic characteristics of the study participants are presented in Table 2

Characteristic	Category	Frequency	Percent (%)
Gender	Male	24	34.3
	Female	45	64.3
	Other	1	1.4
Profession	Doctors	57	81.4
	Nurses	10	14.3
	Allied health professionals	3	4.3
Years of healthcare experience	0–5 years	10	14.3
	6–10 years	17	24.3
	11–15 years	8	11.4
	16–20 years	12	17.1
	21–30 years	14	20.0
	31 + years	9	12.9
Experience with terminally Ill patients	0–2 years	18	25.7
	3–5 years	17	24.2
	6–10 years	16	22.8
	11–20 years	9	12.8
	21–30 years	4	5.7
	31 + years	6	8.5

## Discussion

The results of this study indicate that although healthcare professionals in Pakistan appear to have a moderate awareness and conceptual backing for MAiD, their actual readiness to participate is still restricted. This reluctance seems to be shaped by ethical, legal, and cultural factors. This research is one of very few studies conducted in this region that examine the views of healthcare workers in Pakistan about medical assistance in dying (MAiD), a topic that continues to be ethically controversial and legally unresolved in Pakistan. The results indicate that although participants showed a reasonable understanding of MAiD, their ease or readiness to participate in such practices was restricted. These replies demonstrate a combination of individual, cultural, and systemic factors that still influence the discussion on end-of-life care in Pakistan.

The average knowledge scores shared by participants reflect similar findings from previous research in South Asian settings, where views on euthanasia and physician-assisted suicide are influenced by entrenched religious and cultural beliefs. For example, Abbas et al. (2008) discovered that although Pakistani and Indian doctors were aware of euthanasia, merely a small percentage favored its legalization, with younger and male practitioners showing somewhat more acceptance (Abbas et al. 2008). This indicates that although clinicians might grasp the concept of MAiD, their understanding and ethical perspective are still significantly situational.

Although the individuals in this study demonstrated a substantial theoretical endorsement for the legalization of MAiD, their actual readiness to participate, whether by aiding, directing, or endorsing such actions, was significantly minimal. These results align with information from hospitals in Pakistan, where a significant number of doctors resisted euthanasia and assisted suicide due to ethical issues and religious restrictions (Incardona et al. 2016). Elahi et al. (2024) also emphasize that in Pakistan, healthcare workers frequently have inadequate knowledge and training about MAiD, primarily because of societal stigma, religious limitations, and the lack of public and professional dialogue on this issue. This is in line with the cautious stance that has been observed in clinical settings, where even those who comprehend the justification for MAiD are reluctant to support it due to ethical quandaries or worries about societal backlash. Another possible reason for this reluctance is the limited access to palliative care and basic pain management services across much of the country. When patients’ physical and psychological suffering remains insufficiently addressed, healthcare professionals may feel morally and clinically constrained in endorsing or participating in death-hastening interventions before ensuring that optimal comfort and symptom relief have been achieved.

The analysis revealed significant normative uncertainty, where perceived legal risks and professional vulnerabilities appear to constrain clinical engagement with end-of-life alternatives in the absence of codified MAiD protocols. The absence of clear policy infrastructure actively cultivates this ethical reluctance, as healthcare professionals rationally prioritize self-protection over morally ambiguous clinical innovations (Khan and Siddiqui 2008). This phenomenon reflects a well-documented pattern in developing healthcare systems – where policy gaps translate into clinically restrictive practice norms due to professionals’ vulnerability.

Moreover, the resistance to engage in end-of-life alternatives that include comprehensive palliation may stem from a lack of training, institutional support, and system-level integration of palliative care. Many clinicians have limited exposure to pain management, psychosocial interventions, and interdisciplinary collaboration, which reduces confidence in addressing complex end-of-life needs. In such contexts, even non-controversial approaches like palliative care are perceived as resource-intensive or beyond professional capacity. Thus, reluctance toward MAiD often mirrors broader systemic deficiencies in developing and sustaining holistic end-of-life care models.

Clinical experience, particularly with terminally ill patients, was another significant influence on perceptions of MAiD. Although numerous participants possessed general healthcare experience, many had restricted exposure to end-of-life care. This disconnect might clarify why their perspectives stayed cautious, since regular and significant engagements with terminally ill patients frequently cultivate more compassionate and comprehensive views on end-of-life decisions but also enhanced competence in attending to human suffering. With more exposure, clinicians become better equipped to recognize, interpret, and respond to the physical, emotional, and existential dimensions of distress, which strengthens both their confidence and clinical judgment. According to Khan and Siddiqui’s (2020) recent study, Pakistani caregivers frequently lacked adequate knowledge of palliative care, which not only hindered their ability to assist patients but also affected their confidence in alternative care options (Elmahjub 2019). Enhancing professional education in palliative care will assist healthcare workers in making more knowledgeable and discreet decisions. Thus, expanding professional education and experiential learning in palliative care can help healthcare workers not only make more knowledgeable and discreet decisions but also deliver more effective and compassionate care to those approaching the end of life.

Cultural and religious beliefs could possibly influence perspectives regarding MAiD. In Pakistan, an Islamic country, the value of life is seen as very important, and it is often perceived that factors that contribute to accelerating death are against religious beliefs. The Islamic Code of Medical Ethics emphasizes that human life should be preserved until its natural end, and this principle continues to guide the ethical practices of many Pakistani healthcare professionals (Afzal et al. 2010). Doctors may feel morally obligated to prolong life rather than end it, even in terminally ill patients, which can hinder acceptance of MAiD as an option.

In addition to ethical, legal, and cultural issues, clinical factors significantly influence healthcare providers’ attitudes toward MAiD. The limited availability of palliative care services and lack of access to necessary pain medication in Pakistan create an environment where patients’ suffering is often unaddressed. Without effective symptom management and end-of-life care systems, healthcare professionals may consider MAiD premature ethically and clinically. In fact, nations that have considered or enacted MAiD have concurrently increased palliative care capacity, stressing that extensive care must precede discussions of life-ending interventions.

This study has a few limitations that should be acknowledged. The sample size was relatively small, which may not have captured the full diversity of views among healthcare workers across different institutions. In addition, hospital religious counselors were not included, even though their perspectives could have offered valuable insight into the moral and spiritual aspects of MAiD within Pakistan’s cultural and religious setting. Because the study relied on self-reported responses, some participants may have answered cautiously due to the sensitivity of the topic. Future research that includes a larger and more diverse group of participants, along with voices from spiritual care and policy backgrounds, could help build a more complete understanding of this complex issue.

## Conclusion

In conclusion, healthcare professionals in Pakistan offer a thoughtful and occasionally contradictory perspective on MAiD. Legal ambiguity, religious beliefs, and limited clinical experience in end-of-life care act as barriers to active involvement, despite the presence of some openness and comprehension. These findings emphasize the need for culturally sensitive education, organized training in palliative care, and explicit legal and ethical frameworks to help clinicians manage this complex practice area. Further studies are needed in this area to fully explore the concept, perception, knowledge, and attitude toward MAiD in larger population.

## Appendix

**Section A**: Demographics

Type of health care professional: Doctor/Nurse/Nurse assistant/Physiotherapist

Specialty:

Years of experience in medical profession:

Years of experience dealing with terminally ill patients:

**Section B**: Knowledge and attitude about medical aid in dying

**Knowledge of MAID: Please choose one option for each question**

Yes No I Don’t Know

1. Does MAID require explicit request by the patient?
2. Must the patient suffer from an incurable or incapacitating disease?
3. Is MAID legal anywhere in the world?
4. Is MAID legal in Pakistan?
5. Are there any guidelines available to use for MAID?
6. Is there any circumstance where it is appropriate for a patient to demand MAID?
7. Does MAID come under patient autonomy and Right-to-decide?
8. Is MAID acceptable under any circumstances?
9. Is it the same as Do Not Resuscitate (DNR) orders?
10. Have you ever been asked by a patient to help end their life?

**Attitude toward MAID: Please rate the following statements using your personal beliefs, training, or knowledge**

Strongly Disagree Not Sure Agree Strongly

disagree agree

1.It goes against my religious beliefs

2.It goes against my personal beliefs

3.It goes against the physician oath to preserve life

4.I will be stigmatized for taking part in MAID

5.I will be at risk of legal action if I take part in MAID

6. MAID has an important role in healthcare in the

correct circumstances

7. MAID allows the patient to die with dignity

8. MAID may be legalized in Pakistan with proper ethics

review committees and other safeguards in place

9. > 2 doctors must sign off on the MAID request

10.Psychiatry consult is necessary before accepting MAID

11. MAID is acceptable in grim diagnoses where

life expectancy is limited and the patient requests it

12.Refusal of MAID will make the patient endure

unnecessary physical and mental suffering

13.Inducing death even for merciful reasons is wrong

14.Patient families may be pressured into accepting MAID

15. MAID will be misused by families for a patient

who is severely handicapped

16.Financially poor patients who cannot afford end

of life care may misuse MAID for lack of alternatives

17.A patient denied MAID would find another, more

risky alternative themselves

**Section C**: Clinical Vignette. Please circle yes or no for each question of each patient scenario

Patient vignette **1**: A 30-year-old man suffers from rapidly progressing neuromuscular disease (amyotrophic lateral sclerosis). After 6 months, he can speak only a few simple words, cannot walk, and cannot use his hands to hold objects. He wants to die. Would you directly assist this patient to die? Yes – No

Would you refer this patient to someone to assist him to die? Yes – No

Would it be acceptable for other physicians to assist similar patients to die? Yes – No

Would it be acceptable for non-physicians to assist similar patients to die? Yes – No

Patient vignette **2**: A 60-year-old patient has multiple comorbid, is on dialysis for the past 2 years and is now bedridden after being admitted for a stroke. He previously had his foot amputated due to poorly managed diabetes and his hypertension is poorly controlled. He has developed multiple large pressure ulcers on his back and buttocks, which have become infected with MDR Staph aureus. He does not think he will get any better and wants to die.

Would you directly assist this patient to die? Yes – No

Would you refer this patient to someone to assist her to die? Yes – No

Would it be acceptable for other physicians to assist similar patients to die? Yes – No

Would it be acceptable for non-physicians to assist similar patients to die? Yes – No

Patient vignette **3**: A 70-year-old man suffering from dementia is bedridden after multiple strokes and a previous heart attack, and is in need of 24-hour nursing care. He is from a poor financial background and cannot afford his medications, traveling to the hospital, or nursing care. His family has already spent their life savings on his treatment and cannot continue to care for him.

Would you stop all treatment and let him die? Yes – No

Would you allow the family to stop all treatment and let him die? Yes – No

Would it be acceptable for other physicians to stop all treatment for similar patients to die? Yes – No

Would it be acceptable for the family (or other non-physicians) to stop all treatment? Yes – No

Patient vignette **4**: A 50-year-old woman has suffered from severe pelvic pain for 12 years. The symptoms are thought to be due to depressive and somatoform pain disorders. Eight years of psychotherapy have not helped. The woman refuses antidepressant treatment or electroconvulsive therapy. She wants to die. Would you directly assist this patient to die? Yes – No

Would you refer this patient to someone to assist her to die? Yes – No

Would it be acceptable for other physicians to assist similar patients to die? Yes – No

Would it be acceptable for non-physicians to assist similar patients to die? Yes – No

Patient vignette **5**: The same 50-year-old woman agrees to a complete trial of antidepressants with augmentation and then agrees to electroconvulsive therapy. Both methods of therapy fail to help her and she still wants to die. Would you directly assist this patient to die? Yes – No

Would you refer this patient to someone to assist her to die? Yes – No

Would it be acceptable for other physicians to assist similar patients to die? Yes – No

Would it be acceptable for non-physicians to assist similar patients to die? Yes – No

Vignettes 1, 4, and 5 reproduced with permission from Roberts et al.

**Scoring:**

**Section A: Demographics**

**Section B: Knowledge and Attitude about MAID**

**Scoring**:

- Correct answer = 1 point
- Incorrect answer = 0 points
- “I don’t know” = 0 points

Total Possible Score: 10

Interpretation: Higher scores indicate greater knowledge of MAiD

**Section B: Attitude Questions (17 items)**

- **Scoring**:
  1. Strongly Disagree = 1
  2. Disagree = 2
  3. Not Sure = 3
  4. Agree = 4
  5. Strongly Agree = 5
- Total Possible Score: 85 (17 x 5)
- Interpretation: Scores can be analyzed for:
- Negative attitudes toward MAiD: (1, 2, 3, 4, 5, 13, 14, 15, 16)
- Legal and ethical concerns: (4, 5, 9, 10, 14, 15, 16)
- Support for legalization: (6, 7, 8, 11, 12, 17)

**Section C: Clinical Vignettes**

Each vignette has **4 Yes/No questions** (except Vignette 3, which has **4 different questions**).

- **Scoring:**
  1. **Yes = 1**
  2. **No = 0**
- **Total Possible Score: 20** (5 vignettes x 4 questions each)
- **Interpretation**: Higher scores indicate greater willingness to engage in MAID-related actions.
